# Unveiling Thickness-Dependent Oxidation Effect on Optical Response of Room Temperature RF-Sputtered Nickel Ultrathin Films on Amorphous Glass: An Experimental and FDTD Investigation

**DOI:** 10.3390/ma18122891

**Published:** 2025-06-18

**Authors:** Dylan A. Huerta-Arteaga, Mitchel A. Ruiz-Robles, Srivathsava Surabhi, S. Shiva Samhitha, Santhosh Girish, María J. Martínez-Carreón, Francisco Solís-Pomar, A. Martínez-Huerta, Jong-Ryul Jeong, Eduardo Pérez-Tijerina

**Affiliations:** 1 Centro de Investigación en Ciencias Físico Matemáticas, Facultad de Ciencias Físico Matemáticas, Universidad Autónoma de Nuevo León, Av. Universidad s/n, San Nicolás de los Garza 66455, Nuevo León, Mexico; dylan.huertart@uanl.edu.mx (D.A.H.-A.); ssurabhi@udec.cl (S.S.); maria.martinezcr@uanl.edu.mx (M.J.M.-C.); francisco.solispm@uanl.edu.mx (F.S.-P.); atilano.martinezh@uanl.mx (A.M.-H.); eduardo.pereztj@uanl.edu.mx (E.P.-T.); 2 Departamento de Ingeniería de Materiales (DIMAT), Facultad de Ingeniería (FI), 315 Edmundo Larenas, Box 160-C, Concepción 4070409, Chile; ssaireddy@udec.cl; 3 Drug Delivery Laboratory, Departamento de Ciencias y Tecnología Farmacéuticas, Facultad de Ciencias Químicas y Farmacéuticas, Universidad de Chile, Santiago 8380492, Chile; 4 Nanomaterials and Energy Devices Lab (NMEDL), Department of Mechanical Engineering, NMAM Institute of Technology, Nitte (Deemed to be University), Nitte, Mangalore 574110, Karnataka, India; santhug099@nitte.edu.in; 5 Department of Materials Science and Engineering, Graduate School of Energy Science and Technology, Chungnam National University, Daejeon 305-704, Republic of Korea; jrjeong@cnu.ac.kr

**Keywords:** Ni ultrathin films, natural (ambient) oxidation, RF-sputtering, FDTD simulations, oxidation effect, optical and surficial properties

## Abstract

Nickel (Ni) ultrathin films exhibit phase-dependent electrical, magnetic, and optical characteristics that are significantly influenced by deposition methods. However, these films are inherently prone to rapid oxidation, with the oxidation rate dependent on substrate, temperature, and deposition parameters. The focus of this research is to investigate the temporal oxidation of RF-sputtered Ni ultrathin films on Corning glass under ambient atmospheric conditions and its impact on their structural, surface, and optical characteristics. Controlled film thicknesses were achieved through precise manipulation of deposition parameters, enabling the analysis of oxidation-induced modifications. Atomic force microscopy (AFM) revealed that films with high structural integrity and surface uniformity are exhibiting roughness values (Rq) from 0.679 to 4.379 nm of corresponding thicknesses ranging from 4 to 85 nm. Scanning electron microscopy (SEM) validated the formation of Ni grains interspersed with NiO phases, facilitating SPR-like effects. UV-visible spectroscopy is demonstrating thickness-dependent spectral (plasmonic peak) shifts. Finite Difference Time Domain (FDTD) simulations corroborate the observed thickness-dependent optical absorbance and the resultant shifts in the absorbance-induced plasmonic peak position and bandgap. Increased NiO presence primarily drives the enhancement of electromagnetic (EM) field localization and the direct impact on power absorption efficiency, which are modulated by the tunability of the plasmonic peak position. Our work demonstrates that controlled fabrication conditions and optimal film thickness selection allow for accurate manipulation of the Ni oxidation process, significantly altering their optical properties. This enables the tailoring of these Ni films for applications in transparent conductive electrodes (TCEs), magneto-optic (MO) devices, spintronics, wear-resistant coatings, microelectronics, and photonics.

## 1. Introduction

Plasmon optics or plasmonics achieves extreme light localization and enhanced emission through surface plasmons (SPs), which are light-free electron couplings at metal-dielectric interfaces [1,2]. The coupling of SPs (classified as surface plasmon-polaritons (SPPs) and localized SPs (LSPs)) with electromagnetic (EM) components in metallic nanostructures (NSs) enable to build novel energy, technological, and sensing devices [2,3,4]. Electric and magnetic resonance absorptions in these NSs are the consequence of plasmon hybridization across metallic layers [5,6]. The relative prominence of these plasmon modes is governed by the coupling strength of the hybridization, a process mediated by antisymmetric charge oscillations. Thus, multilayer NSs composed of noble (Au, Ag) and magnetic transition metals (Co, Fe, Ni) exhibit magneto-plasmon mode coupling in their propagating SPPs [7]. The Kretschmann configuration (attenuated total reflection-ATR), which exploits surface plasmon resonance (SPR) and magnetic interactions, is the preferred methodology for achieving advanced sensing capabilities, polarization control, light modulation, and efficient SPP excitation in optical devices [8,9,10]. However, precise thickness control via advanced deposition techniques is crucial for realizing the potential of ultrathin, low-roughness films—comprising diverse materials—in advancing plasmonic device technology.

Noble metal-thin film heterostructures allow for spectral property manipulation [11], and facilitate high-performance SPR characterized by magneto-optical (MO) polarization transformation, magnetic domain interference, and enhanced evanescent field confinement [1,9]. Nevertheless, the morphological driven inherent high SPR quality and low-loss characteristics of noble metals are leveraged through alloying, facilitating wide-range resonance wavelength tunability and optimized field confinement [11,12,13]. On contrary, transition metals offer a viable solution to achieve near-complete conversion with magnetization-dependent phase for applications requiring p-to-s polarization transformation, despite their suboptimal SPR characteristics due to moderate quality and high absorption [9]. This underscores the tunability of plasmonic peak position through incident radiation polarization and external magnetic fields. Particularly, Ni NSs enable the engineering of strong coupling between interband transitions and SPs [14]. Leveraging its thickness and microstructure-dependent optical and electronic characteristics [15], Ni-based materials/NSs facilitate the in-situ realization of self-coupled plasmon-interband polaritons, thereby opening new avenues for controlling light-matter interactions at the nanoscale [16,17,18]. Furthermore, Ni promotes excellent room temperature (RT) ferromagnetism coupled with localized SPR (LSPR) within the UV-visible spectral region, notwithstanding its susceptibility to oxidation and damping [19].

Notably, thickness-dependent surface oxidation of Ni during annealing in ambient environments at elevated temperatures is an inherent process [20]. The inherent susceptibility of Ni to surface oxidation and corrosion presents a significant obstacle to precise thickness control [21]. Specifically, polycrystalline Ni films ranging from 10 to 150 nm in thickness demonstrate accelerated oxidation kinetics between 250 and 500 °C, primarily driven by diffusion along NiO grain boundaries [22]. At higher temperatures (~500 °C), oxide layer growth is governed by the Ni film’s initial grain size and crystallinity, irrespective of preheating, resulting in substantial alterations to the film’s optical and electrical properties [23]. Hence, oxide layer formation is influenced by multiple factors [24,25,26,27], necessitating rigorous control for optimized device performance [28,29,30]. This reveals the critical influence of fabrication methodologies on Ni’s performance regardless of its inherent advantages. Furthermore, the optical repercussions of oxidation on Ni layers require a deeper exploration in tailoring their optical and electrical properties. Consequently, understanding the oxidation and thermal stability of Ni ultrathin films is essential for developing flexible and transparent conductive electrodes (TCEs) [16,18], which can be a potential alternative to indium tin oxide (ITO). Effective mitigation of these limitations necessitates the use of protective, alloying, and usage of buffer layers in conjunction with advanced fabrication techniques within an integrated strategy [11,17,31]. Moreover, the choice of oxidizing agent and fabrication protocol significantly affects the film’s resultant quality and properties [25,32]. RF-sputtering enables the fabrication of Ni ultrathin films with precise control over thickness and optimal growth rates [33,34]. Tailoring the deposition parameters facilitates the production of well-structured films with excellent surface quality.

This study presents a comprehensive investigation of Ni ultrathin films deposited via RT radio frequency (RF) sputtering on Corning glass substrates, examining the natural air oxidation effects on their optical and morphological properties. Film thickness was controlled by deposition time and no external oxidation or thermal annealing was employed on the resultant films. X-ray diffraction (XRD), UV-visible spectroscopy, Scanning Electron Microscopy (SEM), and Atomic Force Microscopy (AFM) unveil the morphological and structural aspects of the fabricated films. Complementary to experimental observations, finite difference time domain (FDTD) simulations were utilized to investigate the influence of thickness-dependent oxidation on Ni optical property modulation, generating detailed information regarding charge and field localizations in both oxidized and unoxidized states. Our analysis indicates that the thickness of the oxide layer profoundly affects the structural, optical, and surface characteristics of RF-sputtered Ni films on glass. Our research establishes a foundation for leveraging the oxide layer to engineer tunable MO and optoelectronic properties of Ni ultrathin films, with applications in next-generation energy harvesting devices.

## 2. Materials and Methods

### 2.1. Experimental—RF Sputtering

**Figure 1:**
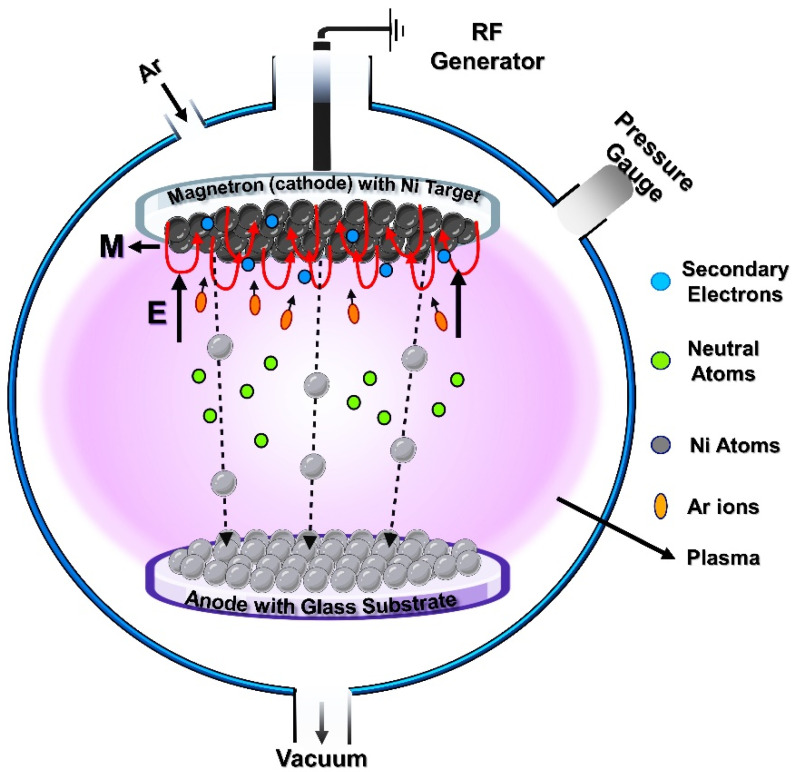
Schematic representation of the RF-magnetron sputtering system.

Figure 1 is the schematic representation of the RF-magnetron sputtering system with which the RT fabrication of Ni ultrathin films on Corning Glass substrate (of size 25 × 75 mm) was carried out. The vacuum chamber was evacuated to an initial pressure of ~5 × 10^−5^ Torr and the deposition was initiated at working power of 100 W under the working pressure of ~4.0–5.0 × 10^−3^ Torr under constant argon (Ar) flow rate of 30 sccm. Here, the Ni target with a purity of 99.99% and Ar gas with a purity of 99.99% were utilized. The distance between the target and the substrate was kept constant at 65 mm. Prior to the original deposition, a pre-sputtering step was carried out for approximately 5 min to remove the surface oxide from the Ni target. The formation of the oxide layer was promoted by atmospheric pressure and RT conditions after deposition. Structural analysis was performed by XRD (PANalytical X’Pert3 Powder) at 45 kV and 40 mA. AFM (Park NX10) was used to analyze the surface texture (thickness and roughness) of thin films. Scanning electron microscopy (SEM) (JEOL JSM-6390LV) is used to analyze the surficial morphology of the samples. Optical transmission studies were investigated using UV-visible spectrophotometers (Thermo Scientific 600 and JASCO V-750) for the wavelength range of 200–900 nm. To ensure the integrity of the films, all samples were prepared in sufficient size to accommodate various characterization techniques simultaneously. Minimal time lapse was allowed to the greatest possible extent between sample removal from the chamber and the first set of characterizations to minimize potential degradation or oxidation.

### 2.2. FDTD Simulation Mechanism

**Figure 2:**
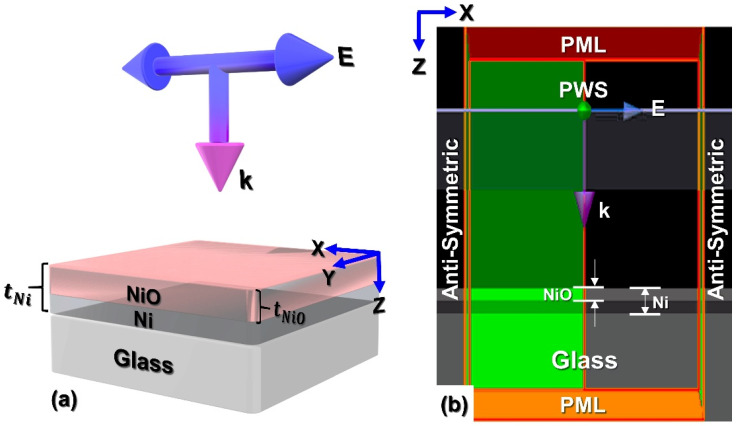
3D FDTD simulation setup of Ni ultrathin films under the illumination of PWS on Glass substrate: (**a**) schematic; (**b**) real simulation environment with relevant boundary conditions.

Thickness-dependent ($t_{Ni}$ = 5–100 nm) optical properties of Ni films irradiated by a plane wave source (PWS) were investigated in FDTD method using Ansys Lumerical module. A Fourier transform analyzed light matter interaction mechanism evaluates the power transmission across the desired spectrum of frequencies. Maxwell’s EM wave equations are solved in three-dimensional (3D) nanostructures (films) using the leapfrog approach by iteratively calculating the future fields in terms of previously calculated fields. The spatial and temporal derivatives of the EM fields were discretized according to the frequencies emitted by the light source. Since all the EM fields are discretized using central-difference approximations in space and time, the spatial and temporal steps are vital parameters to be chosen until the EM field behavior is fully evolved. The vector components of the electric and magnetic fields are spatially staggered about rectangular unit cells of a Cartesian computational grid. Each electric field vector component is located midway between a pair of magnetic field vector components, and vice versa. Figure 2a is the 3D schematic representation of Ni ultrathin films (Kretschmann configuration) placed on glass irradiated by the p-polarized (0°) PWS to probe the $t_{Ni}$-dependent optical absorption through solving the EM wave equations. Here, the E-electric field vector is oscillating along X-axis while the source is propagating (k-vector) along the Z-axis, illuminating the Ni films from their top surface. The magnetic and electric fields are calculated everywhere within the computational domain as a function of time, starting at t = 0. Figure 2b shows the XZ cross-sectional plane of actual FDTD setup in an ambient environment under anti-symmetry (in X, Y-directions) and perfectly matched absorbing layer (PML) boundary conditions (BCs) (in Z-direction), adhering to the symmetry rules, correspondingly. For open boundary problems, absorbing boundary conditions like a PML are used to mimic free space, allowing incident waves and fields to flow through without reflection. As a finite domain numerical method, FDTD requires truncating the computational domain and enforcing proper boundary conditions. For shielded structures, all objects are enclosed within a perfect magnetic or electric conductor box. The numerical stability of the FDTD method’s time marching scheme is a concern. The time step needs to be inversely proportional to the maximum grid cell size to satisfy the Courant–Friedrichs–Lewy (CFL) stability condition. A high-resolution mesh requires a smaller time step, and a smaller time step requires a more significant number of time steps to converge [35]. The frequency dependent complex dielectric constants of the Ni, glass (from in-built library), and NiO [36] are considered accordingly for this evaluation. Section 3.2.1. details the FDTD simulation input parameters employed to investigate the thickness-dependent oxidation effect on the absorbance driven plasmonic peak wavelength modification of Ni ultrathin films deposited on amorphous glass substrates. A systematic variation in thickness parameters was implemented to establish a precise correlation between film thickness and the oxidation-induced plasmonic wavelength shift in Ni ultrathin films. The rationale behind the selection of these parameters is substantiated by the comparative analysis of experimental and simulation results, as evidenced in the respective sections below.

## 3. Results and Discussion

### 3.1. Experimental

#### 3.1.1. Structural Analysis (XRD)

**Figure 3:**
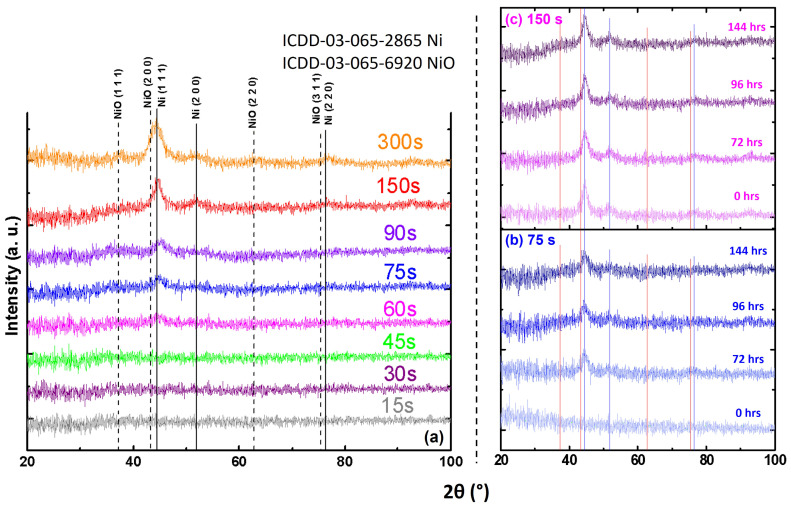
XRD patterns of RF-sputtered Ni ultrathin films on glass substrate (**a**) As-prepared films with different deposition times. The oxidation effect as a function of time lapse (as indicated) for the selected samples of (**b**) 75 s and (**c**) 150 s.

Figure 3a presents the XRD patterns of as prepared RF-sputtered Ni ultrathin films deposited on glass substrates for various durations (15–300 s), resulting in different film thicknesses as outlined in Table 1. The dominant peak observed at 2θ = 44.494° (#ICDD-03-065-2865) indicates Ni preferential growth along the (111) plane with increasing deposition time and film thickness. Additionally, the appearance of NiO peaks at 2θ = 37.442° and 43.473° (#ICDD-03-065-6920) signifies the formation of the oxide phase, particularly in thicker films. To further investigate the oxidation kinetics, two samples with deposition times of 75 and 150 s were exclusively prepared (Figure 3b and Figure 3c, respectively) and characterized at regular time intervals. The 75 s sample, being thinner, exhibits a slower growth rate of oxidation compared to the 150 s sample. The as-prepared 150 s sample clearly shows the presence of both pristine Ni and NiO phases, indicating the adequate growth rate of NiO layer relative to the total film thickness. This confirms that the degree of oxidation (time lapse dependent) and its thickness play a vital role in determining the phase composition and microstructure of Ni thin films, which also influences the optical and electrical properties of the material.

#### 3.1.2. Thickness and Roughness—AFM Analysis

**Figure 4:**
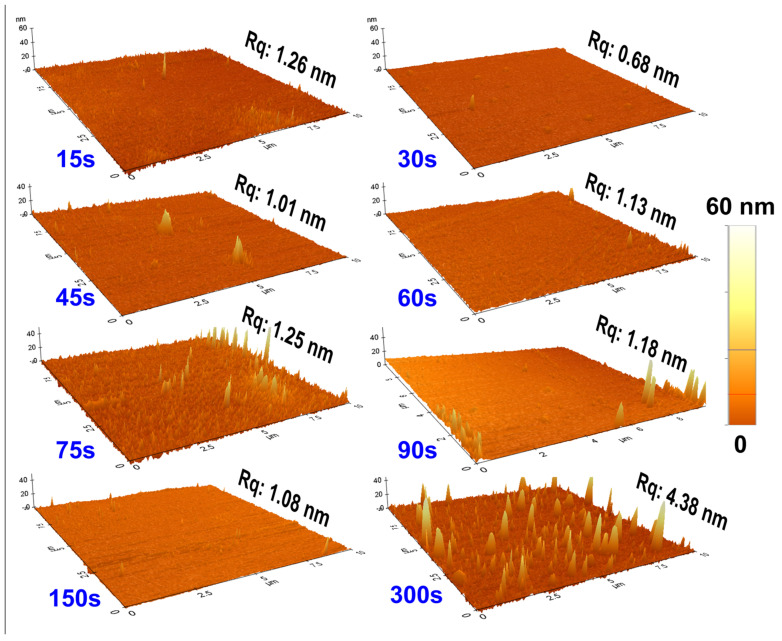
AFM surficial images (10 μm × 10 μm) of as-prepared Ni thin films on glass substrate for different deposition times.

The surface morphology of the as-prepared Ni thin films deposited on glass substrates over an area of 10 μm^2^ was characterized using non-contact mode AFM, as shown in Figure 4. The corresponding root-mean-square (Rq) roughness values for each film are indicated accordingly. All films exhibit a uniform and smooth surface morphology with negligible surface roughness, leveraging high quality structural and morphological aspects. The impact of oxidation on the surface morphology was investigated in 75 s and 150 s samples at regular intervals. The AFM images reveal a uniform growth of the NiO layer on the surface of both samples. The measured areas at similar color scale bars are kept constant for all samples to compare their exceptional surficial quality with oxide layer growth. The discrepancy between the 75 s and 150 s samples’ surface area and negligible initial roughness in Figure S1 (Note S1, Supplementary Information) can be attributed to the measurement area.

**Figure 5:**
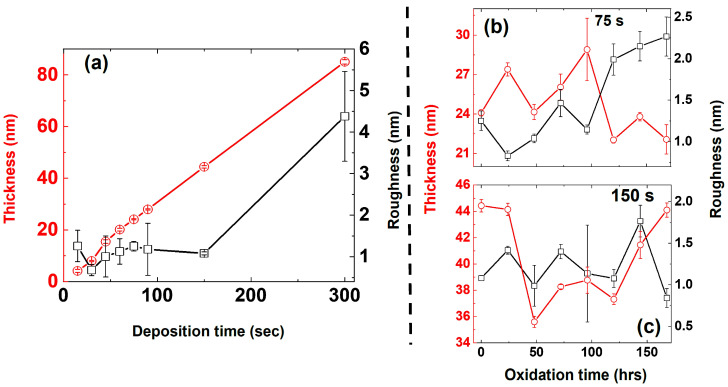
Thickness and roughness vs. deposition time of (**a**) As-prepared Ni ultrathin films and the NiO growth effect for the selected samples of (**b**) 75 s and (**c**) 150 s.

Next, the thickness and Rq values of the films were plotted as a function of deposition (or oxidation) time to further analyze the growth kinetics of the oxide layer and its impact on surface morphology. Figure 5a illustrates the quantitative variation of thickness and Rq values of the as-sputtered Ni films as a function of deposition time, as determined by AFM analysis. These results validate the excellent structural quality of the ultrathin films, with minimal surface roughness. Figure 5b and Figure 5c show the impact of oxidation on the thickness and roughness of the 75 s and 150 s samples, respectively. The active growth rate of the oxide layer and measured area causes slight fluctuations in the net thickness of these films. However, the average thickness (~25 nm for 75 s and ~40 nm for 150 s) and corresponding roughness values (~1.7 nm and 1.55 nm) are consistent with the initial deposition times, indicating a uniform growth of the oxide layer. This further authenticates our notion that the oxide layer significantly influences the surface texture and structural quality of the Ni thin films.

The as-prepared samples with deposition times of 90 s, 150 s, and 300 s, corresponding to grain sizes of approximately ~34, ~41 and ~25 nm, respectively, as determined by XRD, exhibit surface roughness values of ~1.5, ~1 and ~4.5 nm, respectively, as measured by AFM. Samples 75 s and 150 s exhibit the same behavior, with very low Rq values and relatively high grain sizes, as summarized in Table S1 (Note S2, Supplementary Information). These results indicate that the deposited films exhibit a highly homogeneous surface and well-developed granular structure, suggesting optimized film growth.

#### 3.1.3. Optical Properties: Transmittance and Absorbance

**Figure 6:**
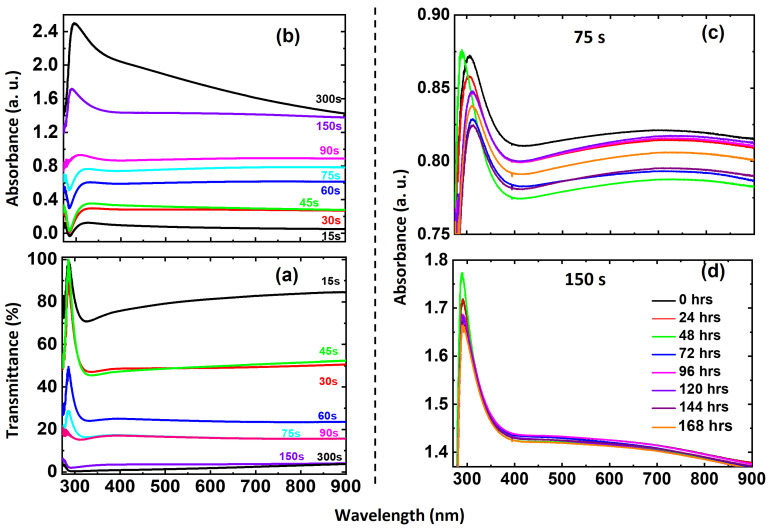
Measured optical (**a**) transmittance and (**b**) absorbance spectra of as prepared Ni ultrathin films with different deposition times. The oxidation effect on optical absorbance as a function of time (as indicated) for the selected samples of (**c**) 75 s and (**d**) 150 s.

Figure 6a,b depict the experimental transmittance and absorbance spectra of as-sputtered Ni ultrathin films with varying deposition times. As expected, thicker films exhibit decreased transmittance and increased absorbance, confirming the thickness-dependent optical response of Ni films. The impact of oxidation on the optical properties of 75 s (Figure 6c) and 150 s (Figure 6d) samples was studied at regular intervals. The 75 s sample shows a significant shift in the absorption peak around 300 nm, while the 150 s sample exhibits a more stable absorption peak position around 290 nm. This difference can be attributed to the relatively faster growth rate of the NiO layer in 75 s sample compared to the 150 s relative to the net Ni layer thickness of both samples.

Optical absorbance increases proportionally with film thickness for all samples, which is consistent with the decrease in transmittance spectra observed across all films. Additionally, it is noted that all films exhibit small average grain sizes, enhancing the scattering of incident light, which thereby reduces the effective absorbance. For the samples deposited for 75 s and 150 s, which were monitored to assess the evolution of their optical absorbance and transmittance properties due to oxidation under atmospheric pressure and RT conditions, UV-vis spectroscopy was used to analyze spectra obtained at various time intervals from 0 to 168 h after deposition. The results indicate that the 75 s deposition sample (~24 nm thick) exhibits the greatest variation in both the maximum absorbance values and their corresponding wavelengths. In contrast, the 150 s deposition sample (~44.5 nm thick) maintains more consistent absorbance values over time, with minimal shifts in both the maxima and their respective wavelengths.

An amorphous structure and/or extremely small grain sizes in thin films can lead to broad and weak diffraction peaks. The characteristics observed in the diffractograms are presented in Figure 3a for the as-deposited samples, with deposition times of 90 s (~28 nm), 150 s (~44.5 nm), and 300 s (~85 nm), which correspond to grain sizes of approximately ~34 nm, ~41 nm, and ~25 nm, respectively. The oxide layer in conjunction with probable defects alters the electrical and optical properties of Ni films. A brief explanation that substantiates the structural mechanisms is given in Note S3, Supplementary Information. In UV-vis spectroscopy, this structural behavior may correspond to the presence of plasmon resonance peaks, as observed in the 90 s, 150 s, and 300 s films.

### 3.2. FDTD Simulations

#### 3.2.1. Thickness-Dependent Optical Analysis—Ni-NiO Thin Films

To further understand the influence of oxidation on the optical properties, FDTD simulations were performed on Ni thin films with thicknesses ranging from 5 to 100 nm. By varying the thickness of the NiO layer while keeping the total bilayer thickness constant (expressed as $t_{Ni}$ in Figure 2), we can isolate the impact of oxidation on the optical response. These simulations provide valuable insights into the relationship between film thickness, oxide layer formation, and optical properties. Table 2 outlines the thickness parameters used in the simulations to evaluate the optical response of pristine Ni films, both with and without variable thicknesses of NiO layer.

#### 3.2.2. Optical Response (Transmittance and Absorbance) of Ni Thin Films–FDTD Simulations

**Figure 7:**
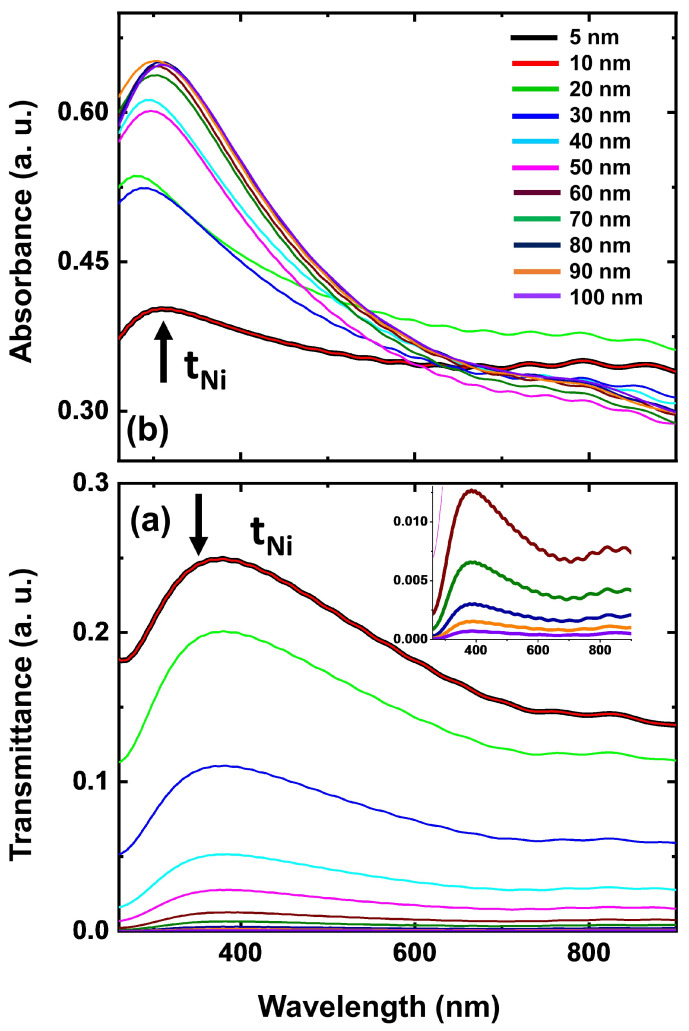
Simulated optical (**a**) transmittance and (**b**) absorbance spectra of Ni ultrathin films of thicknesses ranging from 5–100 nm. (Inset of 7 (**b**) shows the low transmittance observed for the thicker films of thicknesses ranging 60–100 nm).

Figure 7a,b illustrate the simulated transmittance and absorbance spectra of pristine Ni thin films (in the absence of NiO layer) on a glass substrate, considering that the $t_{Ni}$ = 5 to 100 nm as specified in Table 2. Here, Ni films with absolutely no presence of NiO layer were ideally considered to isolate the impact of oxidation on their optical response. This approach was intentionally chosen to accurately comprehend the intrinsic optical properties of the pristine Ni films and to compare the results under the influence of oxidation. A strong correlation is observed between these simulations and experimental results for the nearest corresponding film thicknesses. As expected, the transmittance decreases with increasing film thickness, while the absorbance exhibits an opposite trend. This confirms a linear relationship between absorption and film thickness.

### 3.3. Simulation and Experimental Comparative Analysis:

#### 3.3.1. Thickness Dependence on Plasmonic Peak and Bandgap

**Figure 8:**
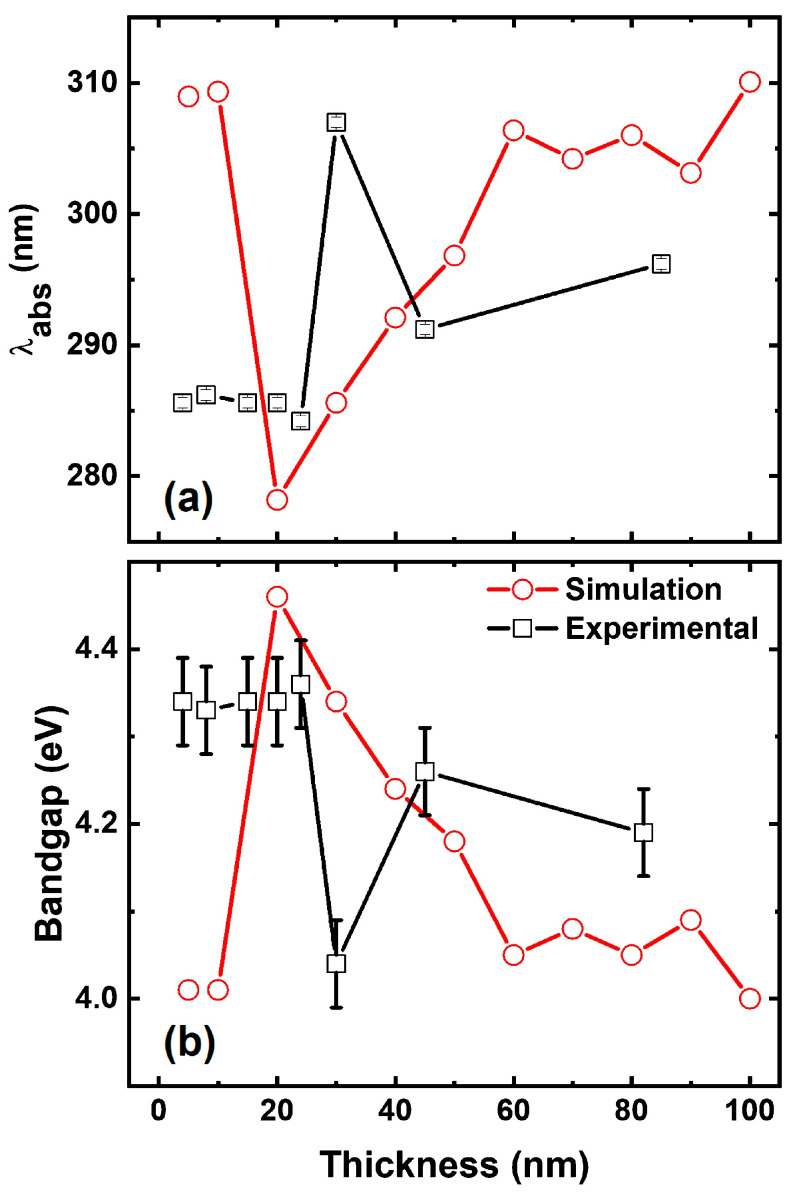
Simulated and experimental variation of thickness dependent (**a**) absorbance peak and (**b**) Bandgap of the Ni thin films without considering the role of NiO layer.

Figure 8a compares the variation of plasmonic absorption peak ($\lambda_{abs}$) positions while Figure 8b matches the energy bandgap (calculated using the empirical relation $E=hc/\lambda_{abs}$) as a function of Ni thicknesses ($t_{Ni}$) based on experimental (Figure 6b) and simulation (Figure 7b) absorptions. Here, E-bandgap (eV) has been calculated using the standard values of h-Planck’s constant (eV-s), c-velocity of light (m/s), and $\lambda_{abs}$. It is assumed that the initial oxidation (takes place upon exposure to ambient environment) has an obvious impact on the experimental determination of $\lambda_{abs}$, and thus the bandgap of the Ni thin films. Nevertheless, it is important to acknowledge that oxidation can instantaneously initiate, and its influence on the optical properties cannot be entirely disregarded. Considering these limitations, we infer that the initial experimental results are primarily reflecting the pristine Ni film properties (with minimal oxidation effects). However, we concede that a minimal degree of oxidation (NiO) inevitably influences the observed plasmonic peak [37]. Experimental and simulated comparative analysis unveils that the bandgap modulation of Ni films is most pronounced within the $t_{Ni}$ = 15–20 nm range (considering NiO contributions) with optimal absorption critically occurring around $t_{Ni}$ = 15 nm [38]. The variation in the bandgap aligns with reported values in the literature considering the potential influence of oxidation [39].

It is noteworthy that the $\lambda_{abs}$ trends exhibit a divergence between experimental and simulated data for $t_{Ni}$ within the 20 to 30 nm range. Experimentally, the plasmonic peak undergoes a redshift from ~285 nm ($t_{Ni}$ = 20 nm) to ~305 nm ($t_{Ni}$ = 30 nm), followed by a blueshift to ~290 nm for thicker films. Conversely, simulations demonstrate a blueshift from ~310 nm ($t_{Ni}$ = 10 nm) to ~275 nm ($t_{Ni}$ = 20 nm), thereby transitioning to a redshift for thicker films. LSPR effects are highly dependent on morphological features, while the dielectric environment surrounding the metallic interface plays a crucial role, which cannot be ignored [40]. The plasmonic absorption bands observed in our Kretschmann configuration are a combination of SPR (~305 nm) [41] and bound electron contributions (<300 nm) [42]. Note S4, Supplementary Information provides a detailed description that supports the plasmonic SPR effect in Ni, which is substantially complemented by the presence of NiO. Despite inconsistencies between experimental and simulated data, resonance activation suggests that the bound electron contribution is probably predominant at the metal-dielectric interface (considering our Kretschmann configuration).

#### 3.3.2. Correlation Between Thickness-Dependent Oxidation and Plasmonic Peak Tunability in Ni-NiO Ultrathin Films—FDTD Simulations

**Figure 9:**
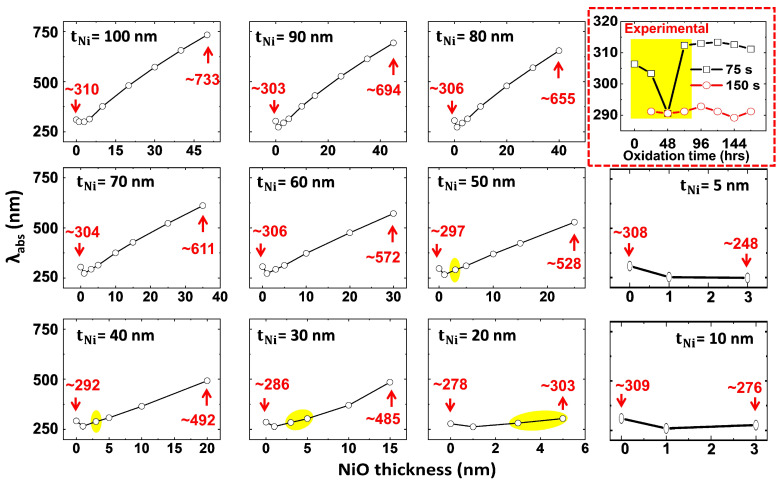
Simulated thickness-dependent oxidation effect on plasmonic peak tunability in Ni-NiO films. The red dotted box illustrates the temporally induced oxidation (NiO layer growth) effect on the plasmonic peak shift of experimentally fabricated 75 s and 150 s Ni film samples for comparative analysis.

A comparative analysis of the oxidation effect on plasmonic absorbance peak position ($\lambda_{abs}$) is presented in Figure 9. The simulated $\lambda_{abs}$ position variation as a function of NiO thickness (Table 2), referencing Figure S2 (Supplementary Information), is compared with experimental data (shown in the red box at the right side) on the plasmonic peak shift due to temporally induced NiO layer growth in 75 s and 150 s Ni films. Correlation between simulated and experimental peak shift data indicates that the introduction of a minimal NiO layer on Ni films leads to a plasmonic peak blueshift relative to pristine Ni films, while a subsequent linear redshift is observed as the NiO layer thickness increases. In the simulation analysis, the initial peak position, corresponding to the absence of NiO (0 nm, Figure 7a), and the final peak position, representing a 50% NiO layer thickness (Figure S2, encompassing the maximum plasmonic shift including second-order peaks), are presented to illustrate the spectral shift as a function of NiO layer growth (expressed in terms of increment in its thickness). It is validated that oxidation significantly modifies the plasmonic absorption peak and enhances broad frequency absorption in Ni films. The extent of this modification, and thus tunability, is highly dependent on Ni-NiO) film’s thickness. Specifically, thin (10 nm) Ni-NiO films show limited peak position variation (5–10 nm per 1–3 nm NiO change), while thicker (100 nm) films demonstrate a significantly larger range (~450 nm per 1–50 nm NiO change).

On the other hand, the temporal evolution of the NiO layer in experimentally fabricated 75 s (thickness ~24 nm) and 150 s (thickness ~44 nm) samples induces distinct plasmonic peak shifts (red circled box in Figure 9). The 75 s sample exhibits an initial blueshift from ~305 nm (0 hrs) to ~290 nm (48 hrs), followed by a subsequent redshift to ~310 nm (48–144 h). The ~305 nm peak position is attributed to the plasmonic resonance of pristine Ni film, contingent upon the absence of NiO [37,41]. Within the Kretschmann configuration and ambient dielectric environment employed in this study, the blueshift to ~290 nm observed during initial NiO growth is attributed to the synergistic contributions of plasmonic interactions, interband electron transitions, and free electron scattering, potentially modulating the NS’s optical response and giving rise to SPR/LSPR-based phenomena [40,42]. To validate these hypotheses, 5 μm area surficial texture SEM scans of the experimentally fabricated samples are presented in Figure S3 (Supplementary Information, Note S6). These scans demonstrate the formation of Ni grains interspersed with NiO phases, which facilitate SPR-like effects. Therefore, we posit that the enhanced localization of EM fields is primarily a consequence of the increased presence of NiO. Subsequently, a progressive increase in NiO layer thickness within the Ni film results in a redshift, yielding a peak position of approximately 310 nm, thus validating the dominant contribution of NiO to the optical properties of Ni-NiO films. Conversely, the 150 s sample shows minimal plasmonic peak shift, maintaining a spectral position within the 290–295 nm range during the observed temporal NiO growth. This behavior implies that the sufficiently thick Ni film (in which NiO presence is predominant), stabilizes the plasmonic peak at ~295 nm owing to the synergistic contributions of plasmonic interactions, interband electron transitions, and free electron scattering.

To ensure correlation between experimental and simulated results, simulated Ni-NiO film thicknesses ($t_{Ni}$) of 20 and 30 nm are compared with the experimentally fabricated 75 s sample, and Ni-NiO films of $t_{Ni}$ = 40 and 50 nm are compared with the 150 s sample, respectively. The plasmonic peak shift observed in the 75 s experimental sample (0–72 h), exhibiting a trend of ~305 > 290 < 310 nm, corresponds well with simulated NiO thicknesses in the range of 3–5 nm. In contrast, the 150 s sample shows a negligible peak shift around ~290 nm (0–72 h), aligning with simulated NiO thicknesses of approximately 3 nm. These findings substantiate the intrinsic influence of NiO on Ni-NiO films and its substantial impact on plasmonic effects in Ni films. To enhance reader convenience, the sections of this study relevant to the comparison are highlighted in yellow. An inherent discrepancy exists between experimental and simulation data due to the idealized conditions of simulations, which disregard surface roughness, interfacial defects, grain size effects, and oxygen diffusion. To gain a deeper understanding of the NiO layer’s maximum effect on the optical response of Ni-NiO films, we present the absorbance trend of Ni films with 50% oxidation (NiO) in Figure S4 (Note S7, Supplementary Information). This highlights the potential exploitation of NiO can enable us to engineer Ni-based films for TCEs and MO applications.

**Figure 10:**
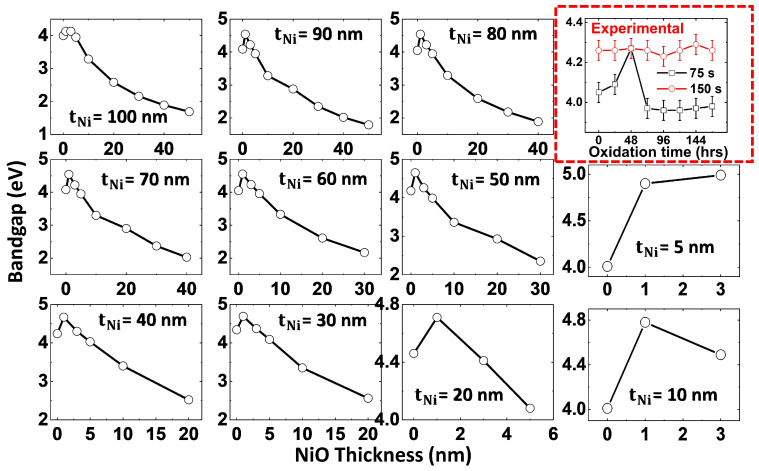
Simulated thickness-dependent oxidation effect on bandgap tunability in Ni-NiO films. The red dotted box illustrates the temporally induced oxidation (NiO layer growth) effect on the variation of bandgap of experimentally fabricated 75 s and 150 s Ni film samples for comparative analysis.

Consistent with the results in Figure 9, Figure 10 presents a comparative analysis of the oxidation effect on bandgap. The simulated bandgap variation as a function of NiO thickness (Table 2), referencing Figure S2 (Supplementary Information), is compared with experimental data (showed in the red box at the right side) on $\lambda_{abs}$ shift due to temporally induced NiO layer growth in 75 s and 150 s Ni films. In accordance with the plasmonic peak shift data, the bandgap trends (both simulation and experimental samples) demonstrate a strong correlation with the progression of NiO growth. This observation validates the significant influence of the oxidation process on the optical properties of Ni thin films. The increased absorption and red-shifted plasmonic peak observed in the experimental data can be attributed to the formation of the NiO layer, which is also supported by the simulation results. These findings unveil that the role of oxidation in Ni ultrathin films can reinforce power absorption efficacy by accommodating tunable optical response. It is possible to optimize the performance of these Ni-NiO films for TEC and MO device applications through precise control over the oxidation process.

### 3.4. Oxidation Induced Electric Field Localization and Self-Heating Effects—FDTD Simulations

**Figure 11:**
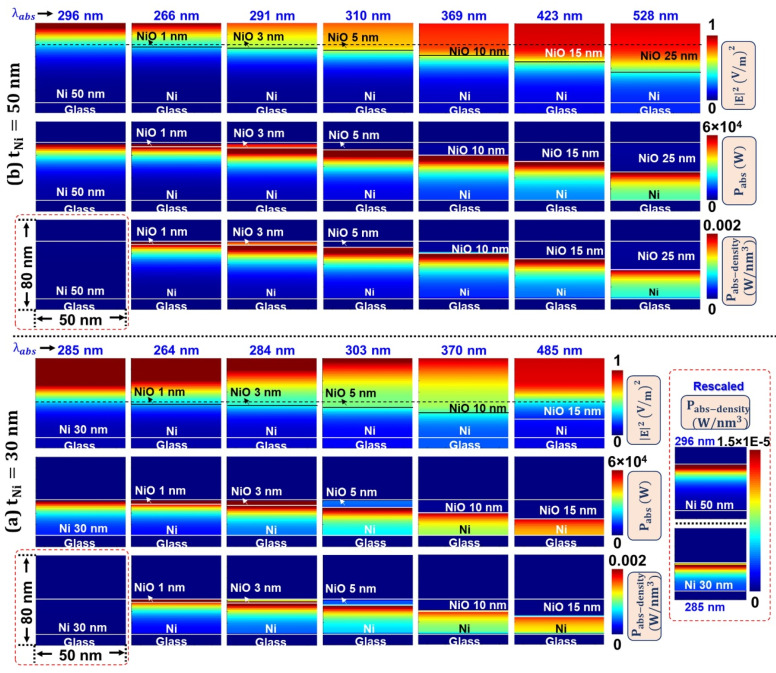
FDTD simulated ${\left|E\right|}^{2}$, $P_{abs}$, and $P_{abs-density}$ profiles in Ni ultrathin films of thicknesses of (**a**) 30 nm and (**b**) 50 nm, with and without a variable thickness of NiO layer.

To validate the proposition that increased NiO presence in Ni-NiO films results in enhanced EM field localization and self-heating, we simulated Electric field intensity (${\left|E\right|}^{2}$), Power absorption ($P_{abs}=\omega \varepsilon_{0}\varepsilon^{\prime \prime }{\left|E\right|}^{2}$), and Power absorption-density ($P_{abs-density}$) profiles for 30 nm and 50 nm Ni films, comparing cases with and without a NiO layer in Figure 11a,b, correspondingly. Here, ω, $\varepsilon_{0}$, $\varepsilon^{\prime \prime }$ are frequency corresponding to the $\lambda_{abs}$ position, permittivity of free space, and relative loss factor, respectively. The stronger dielectric loss factor reflects the greater absorptive capability of the materials. These profiles are captured across the 50 × 80 nm simulated region of the XZ plane shown in Figure 2b. However, the $P_{abs-density}$ is calculated for a 50 × 50 × 80 nm volume and is represented as a 2D XZ-plane plot with scale values that account for the volumetric photothermal response of the system. These results unequivocally unveil the inimitable role of the NiO layer in tuning the $\lambda_{abs}$.

Leveraging its semiconducting properties, the introduction of a NiO layer promotes increased electric field penetration into the Ni film, demonstrating a direct correlation between NiO layer thickness and field localization. Conversely, the absence of NiO restricts EM field propagation, resulting in plasmonic phenomena characteristic of the metal-dielectric interface. Adhering to the correlation between the electric field intensity and power absorption at corresponding plasmonic peak ($P_{abs}=\omega \varepsilon_{0}\varepsilon^{\prime \prime }{\left|E\right|}^{2}$), the absence of an NiO layer results in rapid power absorption, initiated at the metal-dielectric interface. To clarify, we also captured the power absorption density, which shows a very minimal amount of power attenuation, as indicated by the red box (also plotted with rescaled values for clarity).

In contrast, the introduction of a NiO layer (at thicknesses ranging from 1–5 nm) substantially promotes a power absorption gradient (conspicuous with NiO thicknesses 3–5 nm) and augments power absorption density within the Ni layer, manifesting as a redshift in the absorbance peak. This enhanced absorption can be attributed to the synergistic effect between the Ni-NiO bilayers. Notes 3 and 4 in the Supplementary Information elucidate the mechanism by which the oxide layer, in conjunction with probable defect structures, modulates the electrical and optical (plasmonic) properties of Ni films.

Although NiO typically lacks classical SPR, specific NiO structures can exhibit SPR-like behavior under certain conditions, which differ from metallic Ni. A thorough investigation is needed to determine how the room-temperature oxidation diffusion mechanism, which influences NiO’s structure, including grain size effects and interfacial defects, affects its plasmonic properties. This characteristic makes NiO valuable for chemical sensors and optoelectronic devices, where surface sensitivity is crucial. Our findings suggest that the oxidation of Ni thin films can be exploited to utilize them as tunable TCEs and for developing innovative spintronic devices with improved performance. By carefully controlling the thickness of the NiO layer in terms of selecting optimal $t_{Ni}$, it is possible to exploit and tailor the optical properties of Ni thin films-based devices to specific desired applications.

## 4. Conclusions

In this study, Ni thin films with thicknesses in the range of approximately 4 to 90 nm were successfully synthesized on glass substrates using RF-sputtering. XRD, SEM, and AFM characterizations demonstrated the films’ superior structural, morphological, and surface properties. Oxidation growth due to atmospheric pressure and RT conditions on optical response of these films were verified. Experimental data from SEM identified the formation of Ni grains interspersed with NiO phases, correlating with observed SPR-like effects. Complementary UV-visible spectroscopy revealed thickness-dependent spectral shifts, confirming the influence of oxidation and film structure on optical properties. These experimental results were validated by FDTD simulations, which further elucidate the mechanisms at play. Our observations corroborate the thickness-dependent optical absorbance and the resultant plasmonic peak and bandgap shifts. The underlying mechanism for enhanced EM field localization and improved power absorption efficiency is the increased presence of NiO, with the tunability of the plasmonic peak position acting as a key modulator. The oxidation process provides a means to modulate the power absorption characteristics of Ni ultrathin films, opening avenues for their use in TCEs, photonics, and the development of advanced spintronic, MO, wear-resistant, and microelectronic devices.

## Figures and Tables

**Table 1:** RF-sputtered Ni ultrathin films, along with the observed thickness and roughness values for as-prepared samples. Here, thickness and roughness are obtained from AFM analysis while the grain size is calculated from XRD, correspondingly.

S. No.	Deposition Time (s)	Thickness (nm)	Roughness (nm)	Grain Size (nm)
1	15	4.16 ± 0.63	1.26 ± 0.38	-
2	30	7.99 ± 0.29	0.68 ± 0.14	-
3	45	15.48 ± 0.67	1.01 ± 0.50	-
4	60	20.16 ± 0.54	1.13 ± 0.31	-
5	75	24.09 ± 0.23	1.25 ± 0.12	-
6	90	27.96 ± 0.21	1.18 ± 0.63	33.53 ± 1.9
7	150	44.43 ± 0.48	1.08 ± 0.04	41.76 ± 1.2
8	300	85.04 ± 0.88	4.38 ± 1.08	25.83 ± 0.4

**Table 2:** NiO thickness variation with respect to the corresponding total thickness of the Ni film considered for the FDTD simulations.

S. No.	Ni Thickness (nm)	NiO Thickness (nm)
1	5	1, 3
2	10	1, 3, 5
3	20	1, 3, 5, 10
4	30	1, 3, 5, 10, 15
5	40	1, 3, 5, 10, 20
6	50	1, 3, 5, 10, 15, 25
7	60	1, 3, 5, 10, 20, 30
8	70	1, 3, 5, 10, 15, 25, 35
9	80	1, 3, 5, 10, 20, 30, 40
10	90	1, 3, 5, 10, 15, 25, 35, 45
11	100	1, 3, 5, 10, 20, 30, 40, 50

## Data Availability

Data supporting the conclusions of this article will be made available by the authors on request.

## Supplementary Materials

The following supporting information can be downloaded at: https://www.mdpi.com/article/10.3390/ma18122891/s1, Figure S1: Temporal Surface Analysis (10 μm × 10 μm) of Oxidation-Induced Morphological Evolution in 75 s and 150 s Nickel Films on glass substrates.; Table S1: Thickness, roughness, and grain size values of 75 s and 150 s samples obtained from AFM and XRD analyses. Figure S2: Effect of NiO thickness on Ni plasmonic absorbance peak shift. Figure S3: Surficial SEM (of 5 μm) scans of experimentally fabricated samples. Figure S4: (a) Absorbance variation in Ni-NiO thin films at 50% oxidation. (b) Dependence of plasmonic peak variation on the 50% NiO layer thickness relative to the total Ni film thickness. References [43,44,45,46,47,48,49,50,51,52,53,54,55,56] are cited in the Supplementary Materials.

## Abbreviations

The following abbreviations are used in this manuscript:

RF	Radio Frequency
FDTD	Finite Difference Time Domain
SPR	Surface Plasmon Resonance
TCEs	Transparent Conductive Electrodes
MO	Magneto-Optic
SPs	Surface Plasmons
EM	Electromagnetic
SPPs	Surface Plasmon-Polaritons
LSPs	Localized SPs
SAW-FMR	Surface Acoustic Wave-driven Ferromagnetic Resonance
RT	Room Temperature
FM	Ferromagnetic
UV	Ultraviolet
LSPR	Localized Surface Plasmon Resonance
IR	Infrared
XRD	X-ray diffraction
AFM	Atomic Force Microscopy
PWS	Plane Wave Source
PML	Perfectly Matched absorbing Layer
BCs	Boundary Conditions
Rq	Root-Mean-Square
