# Supplementary material for: Unveiling Thickness-Dependent Oxidation Effect on Optical Response of Room Temperature RF-Sputtered Nickel Ultrathin Films on Amorphous Glass: An Experimental and FDTD Investigation
Source: Materials (Basel). 2025 Jun 18;18(12):2891. doi: 10.3390/ma18122891 (PMC12195108; doi:10.3390/ma18122891)
Supplement: Supplementary file 1 [file materials-18-02891-s001.zip › materials-3447239-supplementary.pdf]

Supplementary Information

Unveiling Thickness-Dependent Oxidation Effect on Optical Response of Room Temperature RF-Sputtered Nickel Ultrathin Films on Amorphous Glass: An Experimental and FDTD Investigation

Dylan A. Huerta-Arteaga[1]†, Mitchel A. Ruiz-Robles[1], Srivathsava Surabhi[1, 2]†, S. Shiva Samhitha[2,3], Santhosh Girish[4], María J. Martínez-Carreón[1], Francisco Solís-Pomar[1], A. Martínez-Huerta[1], Jong-Ryul Jeong[5], Eduardo Pérez-Tijerina[1]*

[1] Centro de Investigación en Ciencias Físico Matemáticas, Facultad de Ciencias Físico Matemáticas, Universidad Autónoma de Nuevo León, Av. Universidad s/n, San Nicolás de los Garza, Nuevo León, 66455, México; dylan.huertart@uanl.edu.mx (D. H.); ssurabhi@udec.cl (S. S.); maria.martinezcr@uanl.edu.mx (M. M.); francisco.solispm@uanl.edu.mx (F. S.); atilano.martinezh@uanl.mx (A. M.); eduardo.pereztj@uanl.edu.mx (E. P.)

[2] Departamento de Ingeniería de Materiales (DIMAT), Facultad de Ingeniería (FI), 315 Edmundo Larenas, Box 160-C, Concepción 4070409, Chile; ssurabhi@udec.cl (S. S.); ssaireddy@udec.cl (S.-Sh. S.)

[3] *Drug Delivery Laboratory, Departamento de Ciencias y Tecnología Farmacéuticas, Facultad de Ciencias Químicas y Farmacéuticas, Universidad de Chile, Santiago 8380492, Chile.* ssaireddy@udec.cl (S.-Sh. S.)

[4]Nanomaterials and Energy Devices Lab (NMEDL), Department of Mechanical Engineering, NMAM Institute of Technology, Nitte (Deemed to be University), Nitte – 574 110, Karnataka, India; santhug099@nitte.edu.in (S. G.)

[5] Department of Materials Science and Engineering, Graduate School of Energy Science and Technology, Chungnam National University, Daejeon 305-704, South Korea. ; jrjeong@cnu.ac.kr (J. –R. J.)

* Correspondence: mitchel.ruizrb@uanl.edu.mx (M. R.)

† These authors contributed equally

Note S1: AFM Analysis of 75 s and 150 s Samples

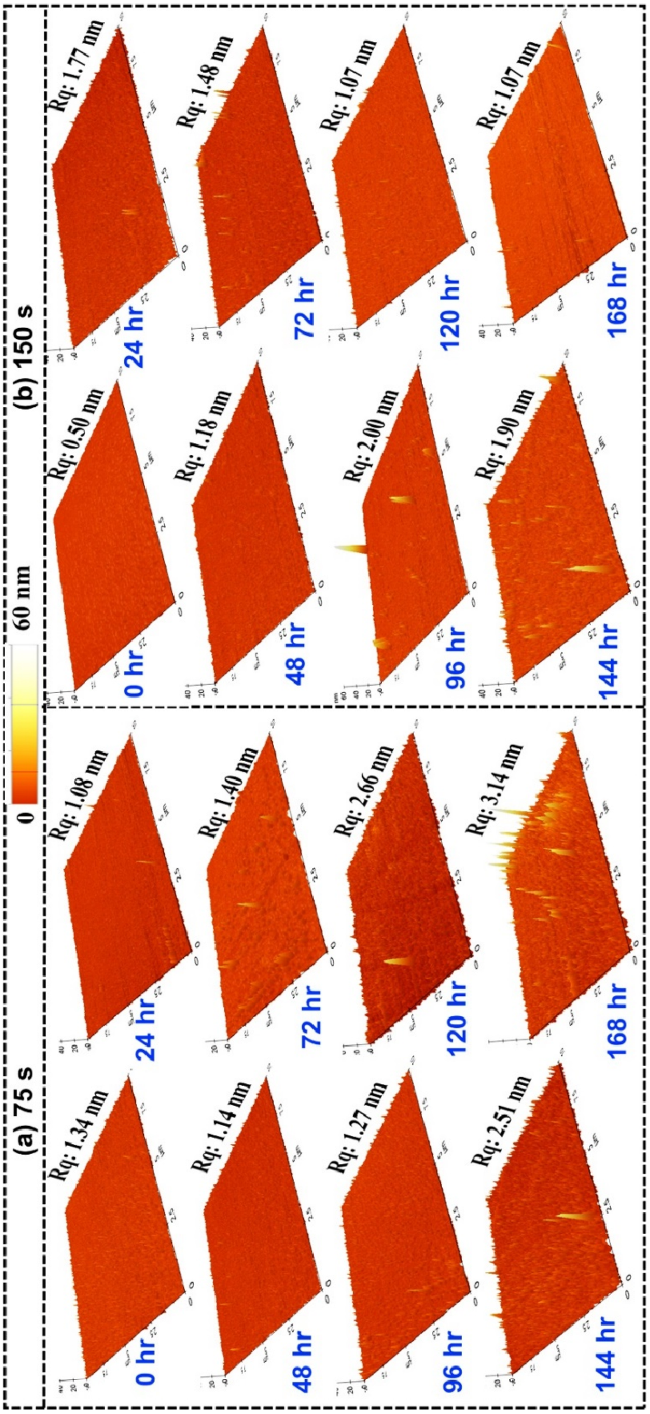

Figure S1. Temporal surface analysis *(10 μm × 10 μm)* of oxidation-induced morphological evolution in 75 s and 150 s Nickel Films *on glass substrates*.

Note S2: Temporal oxidation effect on structural and morphological parameters of 75 s and 150 s samples.

Table S1. Thickness, roughness, and grain size values of 75 s and 150 s samples obtained from AFM and XRD analyses, respectively.

**Oxidation time (hrs)**	**Thickness (nm)**		**Roughness (nm)**		**Grain size (nm)**	
	**75 s**	**150 s**	**75 s**	**150 s**	**75 s**	**150 s**
**0**	24.09 ±0.23	44.43 ±0.48	1.25 ±0.12	1.081 ±0.03	**-**	40.15 ±1.16
**72**	26.05 ±0.99	38.27 ±0.17	1.47 ±0.16	1.40 ±0.09	37.22 ±1.89	40.48 ±1.13
**96**	28.90 ±2.37	38.77 ±1.00	1.15 ±0.05	1.14 ±0.58	35.83 ±2.02	41.64 ±1.15
**144**	23.81 ±0.33	41.45 ±1.03	2.15 ±0.18	1.77 ±0.19	40.69 ±2.53	41.76 ±1.21

Note S3: Nickel film oxidation – Influence of interfacial defects and grain size effects on electrical and optical properties

Interfacial defects originate at the Ni thin film and substrate/NiO layer interface that can substantially alter the electrical and optical properties of the resulting NiO films. Dislocations and grain boundaries can create localized stress fields, affecting carrier mobility and electrical conductivity. Increased defect density augments scattering of charge carriers, resulting in higher resistivity in the NiO films [43]. Oxidation-induced phase separation between NiO and residual Ni can generate a non-uniform phase distribution, which in turn introduces interfacial defects that adversely affect the structural and electronic characteristics of the films [20]. Grain size is another critical factor influencing thin film properties. Smaller grains accelerate oxidation due to increased surface area and reactivity whereas larger grains offer better mechanical strength but less reactivity. Grain size variations severely affect optical transmittance and electrical conductivity due to changes in scattering [44]. Notably, finer grains oxidize faster due to higher surface energy, impacting NiO stoichiometry by potentially increasing the ratio of Ni^{3+} to Ni^{2+}, thus, affecting electronic properties [43,45]. Oxygen diffusion through the nickel film primarily facilitates the reaction with nickel atoms, resulting in the formation of a uniform NiO layer. Temperature, oxygen partial pressure, and film thickness influence the diffusion rate during the diffusion process. Higher temperatures enhance diffusion due to increased atomic mobility [46,47] and promote more complete oxidation but may also induce undesirable microstructural changes like grain coarsening or increased defect density [44,47]. The kinetics of this reaction are influenced by both interfacial defects and grain size. Defects can act as pathways for accelerated oxygen diffusion, while larger grains may hinder this process by reducing the available surface area for reaction [20]. Annealing temperature affects oxygen diffusion during oxidation.

Since no annealing was used in our study, these films are likely independent of annealing-related oxygen diffusion. However, in situ deposition temperatures (in relevance with the 100W power used in this study) may influence the films' stress-strain related growth, subsequently affecting grain size and ambient oxygen diffusion, which demand future investigations on this configuration.

Note S4: Plasmonic effects in Ni/NiO nanostructures

The surface plasmon resonance (SPR) phenomenon is characterized by a distinct minimum in reflected light intensity at a specific angle or wavelength. This resonance shift is sensitive to changes in film thickness, composition, and environmental conditions such as temperature and the presence of analytes. Precisely, the resonance condition for surface plasmon excitation is achieved when the incident light's wave vector matches the surface plasmon wave at the metal-dielectric interface and is highly dependent on the refractive index of the surrounding medium and any variations within the metal layer itself. It is highly sensitive to the refractive indices of the metal/semiconducting and the dielectric medium [48]. Therefore, variations in the dielectric medium's refractive index induce a shift in the resonance angle (θ_{SPR}) or wavelength in the Kretschmann configuration. The successful coupling of incident light into a thin conducting film depends on a precise match between the incident light's wave number and angle and the film's surface plasmon frequency (ω_{SP}) and wave vector (k_{SP}) [49].

When p-polarized light is incident on a metallic/non-metallic thin film at a particular angle, it can excite collective oscillations of free electrons at the metal/dielectric interface, known as surface plasmons. The plasmon frequency (ω_P) described as per the Drude model is as follows

$$\omega_p = \sqrt{\frac{ne^2}{\mu\varepsilon_0}} \qquad (1)$$

Here, n, e, μ, ε_0, are the charge carrier density, charge on the electron, effective mass, and permittivity of the vacuum, respectively. In the Kretschmann configuration, the incident radiation is reflected below the ω, whereas it is transmitted in transparent conducting oxides (like ITO) or absorbed by interband transitions in d-type semiconductors or metals at above ω_P. However, the incident radiation is absorbed and gives rise to the plasmon absorption responsible for SPR effect at the ω_P. A

longitudinal surface plasmon can be excited at the frequency (ω_{SP}) that is defined as $\omega_{sp} = \omega_p/\sqrt{(1 + \varepsilon_s)}$ [50]. Here, ε_s is the dielectric constant of the sample region (in our case it is air; $\varepsilon_s \sim 1$) in contact with the conducting/semiconducting film.

Nickel (Ni) exhibits a high dielectric constant in the infrared region, which significantly contributes to the strong coupling efficiency of surface plasmons (SPR effects). This is attributed to the ability of free electrons in Ni to effectively couple with incident light, resulting in pronounced SPR peaks. Moreover, an increase in the permittivity of the surrounding medium can induce a shift in the resonance condition, thereby altering the SPR response. Unlike metallic Ni films, nickel oxide (NiO) is a p-type wide direct bandgap (3.6-4.2 eV) semiconductor and their thin films do not exhibit a straightforward SPR peak in their absorbance spectra. An undoped NiO is highly resistive (of approximately 10^{13} Ω·cm) at room temperature [51,52].

The polarization response of in case of metal/semiconducting (eg: Ni-NiO) films to an incident electric field, which dictates how electromagnetic waves interact with the material and is quantified as dielectric permittivity, can be expressed as the ratio of the electric displacement field (D) to the electric field (E): $D = \varepsilon E$ [53]. The dielectric permittivity of NiO is highly variable, influenced by factors such as strain, temperature, and intrinsic film defects [54]. Research demonstrates that strained NiO films exhibit an elevated dielectric permittivity, primarily attributed to space-charge polarization mechanisms, particularly at elevated temperatures. This increased dielectric permittivity can enhance SPR sensitivity by facilitating more efficient coupling of surface plasmons with incident light. This enhancement is further augmented by contributions from electronic and ionic polarization mechanisms [53].

In our study, the Ni films produced via RF sputtering exhibited spontaneous oxidation when exposed to the ambient atmosphere. Conventional UV-visible studies under normal incidence of light is considered in this study. Consequently, the transition from metallic (Ni) to semiconducting (NiO) behavior significantly impacts their optical response. A detailed analysis of the resonance absorbance peak shifts observed in both pristine Ni and Ni-NiO films reveals the following: the prominent resonance peak characteristic of Ni appears at a lower wavelength, a consequence of its higher free electron density associated with its metallic energy bands. Conversely, the redshift of the comparatively weak NiO plasmonic peak is attributable to its lower electron density and semiconducting nature. Furthermore, the plasmonic peak intensity of Ni films surpasses that of NiO films, reflecting the stronger coupling of surface plasmons with incident light in metallic materials compared to semiconducting materials like NiO. This difference in intensity may also be influenced by broader absorption features in NiO, resulting from bandgap transitions.

Recent research has demonstrated that specific forms of NiO, notably two-dimensional amorphous NiO can exhibit plasmonic behavior under carefully controlled conditions. Contrary to the typical association of SPR with metals, some conductive metal oxide morphologies such as NiO have demonstrated SPR-like properties. This phenomenon arises from their capacity to support collective electron oscillations, albeit with distinct attributes compared to those observed in metals [49]. In response to electromagnetic waves, conduction electrons within these materials undergo oscillations, generating a surface electric field with a limited penetration depth (skin depth). Furthermore, structural and compositional modifications have been shown to induce morphological influences on two-dimensional amorphous NiO, revealing its potential as a plasmonic photocatalyst. This is evidenced by an absorbance peak, indicative of SPR, at approximately 529

nm, suggesting that specific structural arrangements can confer plasmonic properties to NiO [55]. It is important to note that while NiO thin films demonstrate nonlinear optical properties, which may be observed alongside SPR-like phenomena. These properties are generally distinct from the conventional SPR characteristic of metals [56].

In summary, while classical SPR is not a typical characteristic of NiO thin films, specific structural forms can exhibit plasmonic behavior under particular conditions. The plasmonic peak characteristics observed in these instances can differ significantly from those observed in Ni metallic films. This phenomenon necessitates future studies focused on elucidating the structural influence on plasmon-induced SPR-like properties of NiO formed through room-temperature fabrication and spontaneous oxidation of nickel under ambient atmospheric conditions. This characteristic renders NiO particularly effective for applications in chemical sensors and optoelectronic devices, where the ability to detect subtle environmental changes or chemical interactions at the surface is paramount.

Note S5: Thickness Dependence Oxidation of Nickel Thin Films – FDTD Simulations

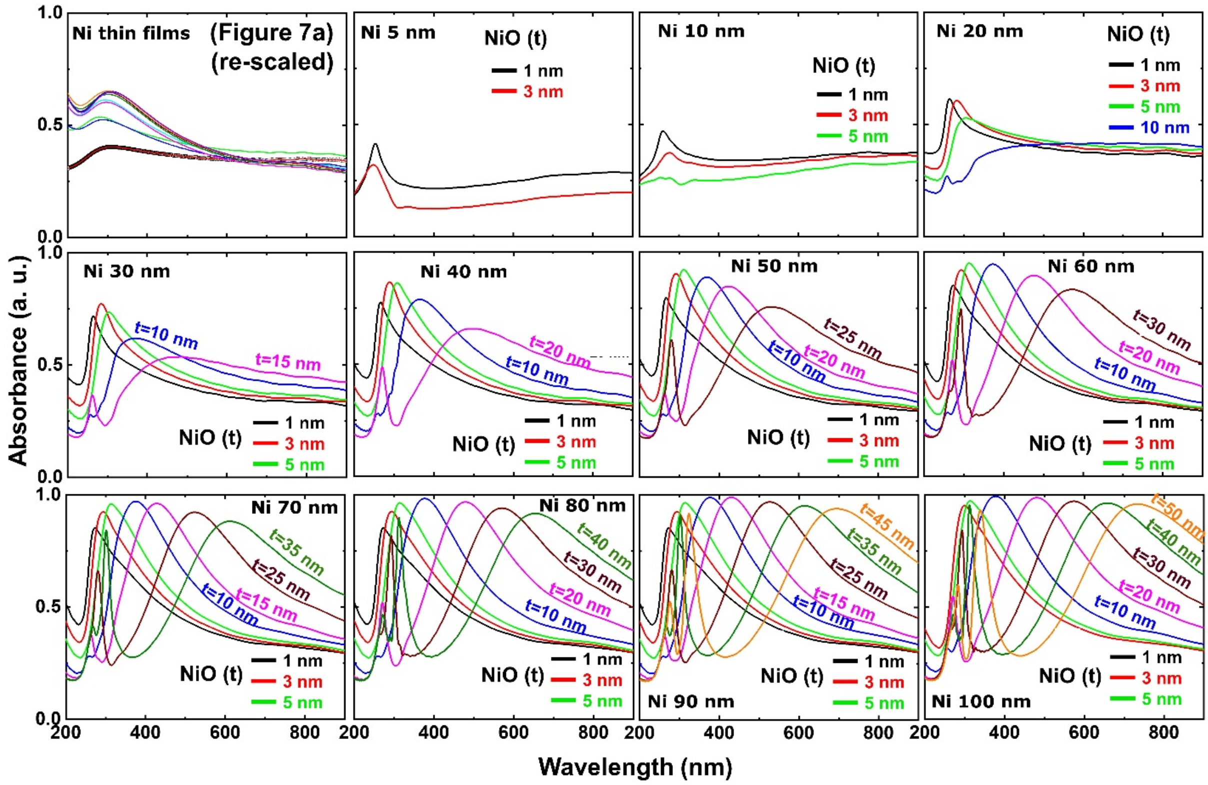

Figure S2. Effect of NiO thickness on Ni plasmonic absorbance peak shift.

To explore the NiO layer role on plasmonic peak shift and optical properties of Ni films, we conducted a detailed FDTD-based investigation into the influence of oxidation on the optical properties of Ni thin films, across a range of thicknesses from ultrathin to bulk. By controlled variation of the NiO layer thickness (up to 50% of the total film thickness as indicated in **Table 2** – main manuscript), we determined the effects of oxidation on these optical properties. **Figure S2** presents the simulated absorbance spectra of Ni films with varying NiO layer thicknesses, juxtaposed with the spectrum of pristine Ni ultrathin films (**Figure 7a**). The data demonstrate that increment in the NiO layer thickness significantly alters the absorption spectra and plasmonic peak (λ_{abs}) position, including a shift in the absorption maximum and the

emergence of a second-order resonance. Furthermore, the overall absorbance across the UV-visible frequency spectrum increases with the existence of the NiO layer over Ni film. These results are consistent with our previous observations (**Figures 6-8** – main manuscript), which established that: (i) increased total film thickness enhances absorption, (ii) films within the 15-20 nm total thickness range exhibit substantial variations in net absorbance compared to thicker films, and (iii) the bandgap is sensitive to the Ni film's oxidation state.

Note S6: Morphological Analysis of Nickel Thin Films – SEM

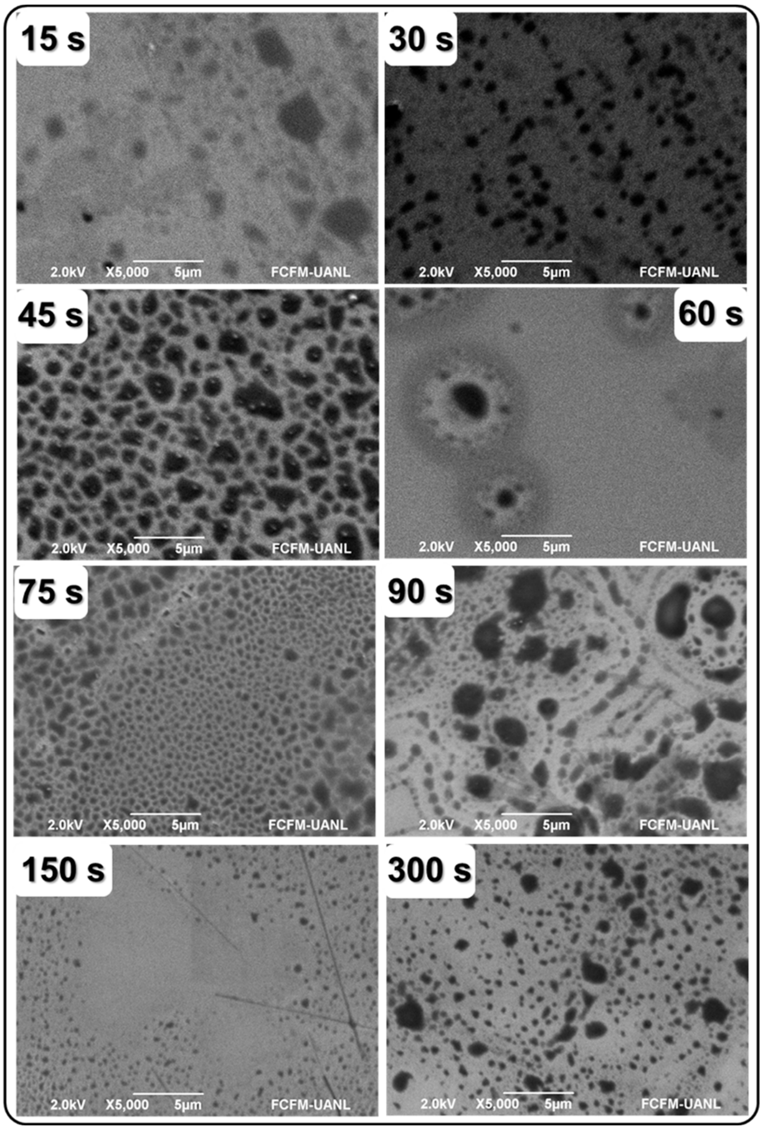

Figure S3. Surficial SEM (of 5 μm) scans of experimentally fabricated samples in this study.

Note S7: Spectral absorbance variation in Ni-NiO thin films with a 50% NiO thickness – FDTD Simulations

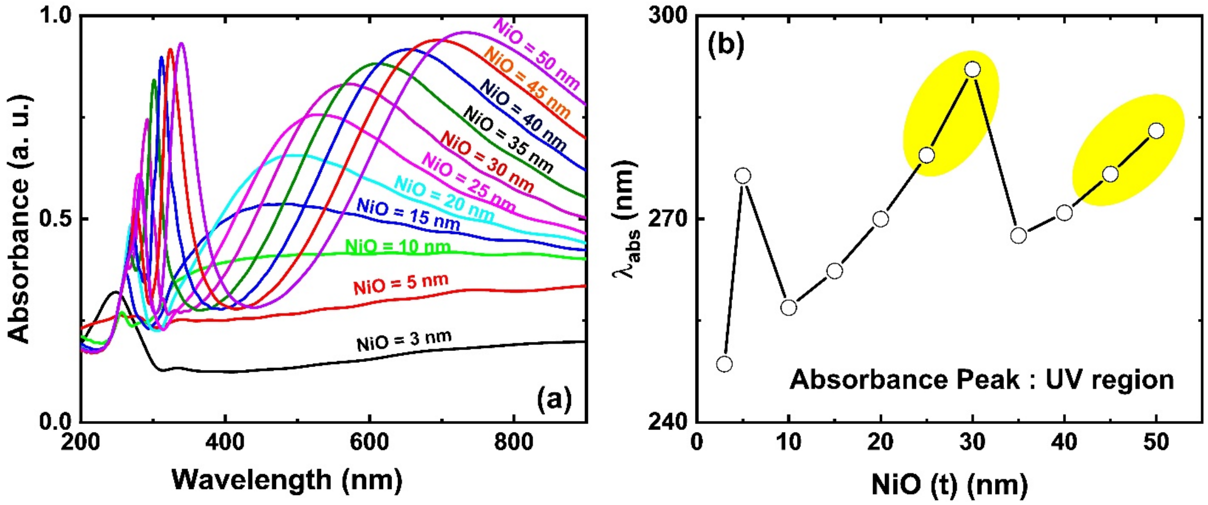

Figure S4. (a) Absorbance variation in Ni-NiO thin films at 50% oxidation. (b) Dependence of plasmonic peak variation on the 50% NiO layer thickness relative to the total Ni film thickness.

Figure S4a presents a comparative analysis of absorbance variation in Ni-NiO thin films with maximum oxidation (50% of total film thickness). **Figure S4b** depicts the spectral variation of the first plasmonic peak in the UV region as a function of NiO layer thickness, demonstrating its dependence on the total film's thickness. The observed data validates our analysis presented in **Figure 9** (main manuscript – experimental subsection, 75 s and 150 s samples), indicating that a 3-5 nm NiO layer effectively modulates the plasmonic peak position within the UV spectral range of Ni-NiO films, where the inherent plasmonic resonance occurs. It is crucial to differentiate that the maximum plasmonic peak wavelengths presented in Figure 9 (simulation results) pertain to the second-order plasmonic resonance in the visible spectral range but not the UV region. To facilitate reader comprehension, the comparison thicknesses are indicated with yellow highlighting.

References

Note: Reference numbers correspond to the order in they appear in main manuscript. Please consult the main manuscript for full reference details.

20. Ravikumar, P.; Taparia, D.; Alagarsamy, P. Thickness-Dependent Thermal Oxidation of Ni into NiO Thin Films. J. Supercond. Nov. Magn. 2018, 31, 3761–3775. https://doi.org/10.1007/S10948-018-4651-6

43. T.K.H. Pham, B.Q. Tran, K.B. Nguyen, N.Y.N. Pham, T.H.Y. Nguyen, A.H.T. Nguyen, N.P. Nguyen, H.D. Ngo, H.P. Pham, Oxygen partial pressure effects on nickel oxide thin films and NiO/Si diode performance, Mater. Adv. 6 (2025) 1719–1725. https://doi.org/10.1039/D4MA01113A.

44. S. Barala, S.A. Panda, S. Gangopadhyay, Growth and Characterization of NiO Thin Films for Selective Detection of Formaldehyde Vapor, Phys. Status Solidi A. (2024) 2400695. https://doi.org/10.1002/pssa.202400695.

45. F. A. F. Lahiji, S. Bairagi, R. Magnusson, M.A. Sortica, D. Primetzhofer, E. Ekström, B. Paul, A. le Febvrier, P. Eklund, Growth and optical properties of NiO thin films deposited by pulsed dc reactive magnetron sputtering, J. Vac. Sci. Technol. A. 41 (2023) 063402. https://doi.org/10.1116/6.0002914.

46. M. Benedet, C. Maccato, G. Pagot, C. Invernizzi, C. Sada, V. Di Noto, G.A. Rizzi, E. Fois, G. Tabacchi, D. Barreca, Growth of NiO Thin Films in the Presence of Water Vapor: Insights from Experiments and Theory, J. Phys. Chem. C. 127 (2023) 22304–22314. https://doi.org/10.1021/acs.jpcc.3c05067.

47. S. Timoshnev, A. Kazakin, K. Shubina, V. Andreeva, E. Fedorenko, A. Koroleva, E. Zhizhin, O. Koval, A. Kurinnaya, A. Shalin, V. Bobrovs, Y. Enns,

Annealing Temperature Effect on the Physical Properties of NiO Thin Films Grown by DC Magnetron Sputtering, Adv. Mater. Interfaces. 11 (2024) 2300815. https://doi.org/10.1002/admi.202300815.

48. S.A. Mahmoud, A. Shereen, M.A. Tarawnh, S.A. Mahmoud, A. Shereen, M.A. Tarawnh, Structural and Optical Dispersion Characterisation of Sprayed Nickel Oxide Thin Films, J. Mod. Phys. 2 (2011) 1178–1186. https://doi.org/10.4236/jmp.2011.210147.

49. C. Rhodes, S. Franzen, J.P. Maria, M. Losego, D.N. Leonard, B. Laughlin, G. Duscher, S. Weibel, Surface plasmon resonance in conducting metal oxides, J. Appl. Phys. 100 (2006) 054905. https://doi.org/10.1063/1.2222070.

50. F. Wooten, Optical properties measurement techniques solar energy materials special issue, Academic Press, New York, NY, USA; London, UK, 1972.

51. C. Zaouche, S. Benramache, A. Gahtar, Electrical and Magnetic Properties of NanostructuredNiO Thin Films Prepared by Spray Pyrolysis Method, Biomed. J. Sci. Tech. Res. 52 (2023) 43761–43766. https://doi.org/10.26717/BJSTR.2023.52.008258.

52. A.T. Oluwabi, N. Spalatu, N. Maticiuc, A. Katerski, A. Mere, M. Krunks, I.O. Acik, Combinative solution processing and Li doping approach to develop p-type NiO thin films with enchanced electrical properties, Front. Mater. 10 (2023) 1060420. https://doi.org/10.3389/fmats.2023.1060420.

53. A. Kashir, H.W. Jeong, G.H. Lee, P. Mikheenko, Y.H. Jeong, Dielectric Properties of Strained Nickel Oxide Thin Films, J. Korean Phys. Soc. 74 (2019) 984–988. https://doi.org/10.3938/jkps.74.984.

54. D. Abubakar, N.M. Ahmed, S. Mahmud, Structural, Electrical and Optical Properties of NiO Nanostructured Growth Using Thermal Wet Oxidation of Nickel Metal Thin Film, J. Nano Res. 49 (2017) 56–65. https://doi.org/10.4028/www.scientific.net/JNanoR.49.56.

55. Z. Lin, C. Du, B. Yan, C. Wang, G. Yang, Two-dimensional amorphous NiO as a plasmonic photocatalyst for solar H2 evolution, Nat. Commun. 9 (2018) 4036. https://doi.org/10.1038/s41467-018-06456-y.

56. Bhakta, R. Panda, P.K. Sahoo, Third-order non-linear optical switching and threshold limiting of Nio thin films, Sci. Rep. 14 (2024) 22767. https://doi.org/10.1038/s41598-024-69853-y.
